# *Vibrio cholerae* O139 persists in Dhaka, Bangladesh since 1993

**DOI:** 10.1371/journal.pntd.0009721

**Published:** 2021-09-02

**Authors:** Irin Parvin, Abu Sadat Mohammad Sayeem Bin Shahid, Subhasish Das, Lubaba Shahrin, Mst. Mahmuda Ackhter, Tahmina Alam, Soroar Hossain Khan, Mohammod Jobayer Chisti, John D. Clemens, Tahmeed Ahmed, David A. Sack, Abu Syed Golam Faruque

**Affiliations:** 1 Nutrition and Clinical Services Division, International Centre for Diarrhoeal Disease Research, Bangladesh (icddr,b), Dhaka, Bangladesh; 2 Office of the Executive Director, International Centre for Diarrhoeal Disease Research, Bangladesh (icddr,b), Dhaka, Bangladesh; 3 UCLA Fielding School of Public Health, Los Angeles, California, United States of America; 4 Department of International Health, Johns Hopkins University Bloomberg School of Public Health, Baltimore, Maryland, United States of America; Christian Medical College, Vellore, INDIA

## Abstract

**Background:**

After a multi-country Asian outbreak of cholera due to *Vibrio cholerae* serogroup O139 which started in 1992, it is rarely detected from any country in Asia and has not been detected from patients in Africa.

**Methodology/Principal findings:**

We extracted surveillance data from the Dhaka and Matlab Hospitals of International Centre for Diarrhoeal Disease Research, Bangladesh (icddr,b) to review trends in isolation of *Vibrio cholerae* O139 in Bangladesh. Data from the Dhaka Hospital is a 2% sample of > 100,000 diarrhoeal patients treated annually. Data from the Matlab Hospital includes all diarrhoeal patients who hail from the villages included in the Matlab Health and Demographic Surveillance System. *Vibrio cholerae* O139 was first isolated in Dhaka in 1993 and had been isolated every year since then except for a gap between 2005 and 2008. An average of thirteen isolates was detected annually from the Dhaka Hospital during the last ten years, yielding an estimated 650 cases annually at this hospital. During the last ten years, cases due to serogroup O139 represented 0.47% of all cholera cases; the others being due to serogroup O1. No cases with serogroup O139 were identified at Matlab since 2006. Clinical signs and symptoms of cholera due to serogroup O139 were similar to cases due to serogroup O1 though more of the O139 cases were not dehydrated. Most isolates of O139 remained sensitive to tetracycline, ciprofloxacin, and azithromycin, but they became resistant to erythromycin starting in 2009.

**Conclusions/Significance:**

Cholera due to *Vibrio cholerae* serogroup O139 continues to cause typical cholera in Dhaka, Bangladesh.

## Author summary

*Vibrio cholerae* O139 isolates were reported for the first time in Dhaka in 1993, but then declined and seemed to disappear as a threat. It was never isolated from patients in Africa. Data from the diarrhoeal diseases surveillance systems of hospitals in Dhaka and Matlab, Bangladesh were reviewed to observe the trends in isolation of *Vibrio cholerae* O139. Based on a representative 2% sample of patients at the Dhaka hospital, about 650 cholera patients were infected with *V*. *cholerae* O139 in recent years except during a gap period between 2005 and 2008 when no isolates were identified. The illnesses due to serotype O139 represent less than 1% of all cholera patients seen at the Dhaka hospital. The last patient with cholera due to *V cholerae* O139 in Matlab was detected in 2005. Clinical signs and symptoms of cholera due to serogroup O139 and serogroup O1 were similar though more of the O139 cases were not dehydrated. Most isolates of O139 remained susceptible to tetracycline, azithromycin, and ciprofloxacin; however, sensitivity to erythromycin changed following the gap years. Prior to 2005, they were sensitive, but they were resistant when they returned in 2009. We conclude that cholera caused by *Vibrio cholerae* O139 has not disappeared but continues at a low level in Dhaka, Bangladesh and surveillance needs to continue for this serogroup in Bangladesh.

## Introduction

Cholera is a severe dehydrating diarrhoeal disease caused by intestinal infection with *Vibrio cholerae* which spreads in epidemics causing considerable mortality and morbidity in Africa, Asia, and Hispaniola [1]. Being primarily transmitted through contaminated water, it occurs most often in areas with contaminated water and inadequate sanitation [2]. Since the early 1800s, there have been seven cholera pandemics in which *V*. *cholerae* has spread through multiple countries and regions [2]. Although there are more than 200 identified serogroups of *V*. *cholerae*, toxigenic strains of *V*. *cholerae* serogroup O1 were responsible for pandemic cholera [3]. In late 1992 however, an outbreak of cholera began in Madras and then spread all over India, and the southern part of Bangladesh due to a serogroup that was not O1, but rather toxigenic serogroup O139, subsequently known as O139 Bengal [4, 5]. This was a dramatic change in the understanding of cholera’s epidemiology since previous epidemics were caused only by serogroup O1. This new serogroup spread widely in Asia for a period of a few years but did not enter Africa as previous strains of serogroup O1 had done. Then, after this large outbreak in the early 1990s, the numbers of cases decreased and *V*. *cholerae* O139 strain is rarely isolated from clinical samples [6] and countries have not reported it to the World Health Organization (WHO) since 2014 [7]. The most recent published reports of clinical cases due to serogroup O139 were from India [8], Bangladesh [3] and China [9]. In spite of not being reported from other areas, cases due to *V*. *cholerae* O139 are still being seen in the Dhaka Hospital of the International Centre for Diarrhoeal Disease Research, Bangladesh (icddr,b) in Dhaka, Bangladesh.

Bangladesh is a country with endemic cholera due to serogroup O1. In the middle part of the country, including Dhaka, there are two seasonal peaks [10]. Bangladesh does not have a national cholera surveillance system but more than 100,000 cases, including about 3000 deaths, occur annually [1, 11]. The ecosystem of flooding in the Ganges Delta, plus the poorly developed sanitation and public health system of Bangladesh limits the ability to reduce human exposure to this ubiquitous organism.

The present study was designed to determine the trends of cholera, describe the baseline and clinical characteristics of cholera cases caused by *V*. *cholerae* O139 and *V*. *cholerae* O1 serogroups, and trends in antibiotic susceptibility among clinical isolates of *V*. *cholerae* O139 serogroup obtained during 1993–2020 in an urban diarrhoeal disease hospital in Dhaka, Bangladesh and in a rural hospital at Matlab, Bangladesh.

## Materials and methods

### Ethics statement

We obtained verbal informed consent from the participants or their parents or legal guardians before enrolling them into the study. The institutional review boards (IRB; named as Research Review Committee and Ethical Review Committee) of icddr,b reviewed and approved the study protocol.

### Study design

The study used data from facility-based surveillance systems that monitored patients who sought care for diarrhoeal diseases in the urban Dhaka Hospital and the rural Matlab Hospital of icddr,b. We have included data from diarrhoeal patients enrolled in the DDSS of Dhaka Hospital, most of whom come from the urban and peri-urban areas of Dhaka city. The data from the Matlab Hospital also includes all diarrhoeal patients who hail from the villages into the Matlab Health and Demographic Surveillance System (HDSS) of icddr,b.

These specialized research and training health facilities primarily provide care to patients with diarrhoeal illnesses or acute respiratory infections with or without associated complications and health problems. Most of the patients are from the poor socioeconomic strata. Since inception, the facilities provide free-of-cost care to the patients and, for the last 10 years, 140,000–190,000 patients annually were treated in the Dhaka Hospital. 18,000–48,000 patients were treated annually at the Matlab Hospital, but only about 3–7% of them live in the HDSS area.

In the Dhaka Hospital, since 1979, icddr,b had been operating DDSS that systematically samples patients to collect demographic, socioeconomic, and clinical information using a standard structured questionnaire. This included a 4% sample from 1979 through 1995; then a 2% sample since 1996. At the Matlab Hospital, data from patients seeking care were recorded in the surveillance system, if they live in villages as defined in the HDSS area. In 2014, the population of the 142 villages in the HDSS was 230,185 [12].

Demographic, socioeconomic, clinical, and laboratory data collected from all patients enrolled in the DDSS of Dhaka Hospital of icddr,b between January 1993 (the first year when *Vibrio cholerae* O139 was detected in Dhaka) to December 2020 were utilized to form the analyzable dataset for the Dhaka Hospital. Data from 2000 to 2020 was included from the Matlab Hospital. Since socioeconomic indicators have changed over nearly three decades of this surveillance period, the socioeconomic data as well as the clinical features during the last ten years (2011 to 2020) were used when comparing these factors.

Data were included from diarrhoeal patients (three or more loose stools per 24 hours) of all age groups who were treated in the Dhaka Hospital of icddr,b and enrolled in the DDSS, or diarrhoeal patients who were treated at the Matlab Hospital and lived in the villages included in the HDSS system during the study period.

### Laboratory methods

Recently collected stool specimens from all enrolled patients were transported to the clinical microbiology laboratories of the respective hospitals. In Dhaka Hospital, stool samples were routinely screened for common enteric pathogens including *V*. *cholerae*, Enterotoxigenic *Escherichia coli* (ETEC), *Shigella spp*., *Salmonella spp*. and rotavirus [13]. At the Matlab Hospital laboratory diagnoses were carried out to isolate *V*. *cholerae*, *Salmonella spp*. *and Shigella spp*. Isolation, identification, serogrouping, and biotyping of these bacterial pathogens were performed using standard laboratory procedures. Antimicrobial susceptibility was determined by the standard disc diffusion method on Muller-Hinton agar with commercial discs (BD, Becton, Dickinson, and Company, USA) and the interpretative categories of sensitive, intermediate, and resistant were determined based on the cutoff of the zone size for antibiotics according to the up-to-date Clinical and Laboratory Standards Institute guidelines for *V*. *cholerae* [14].

### Data analysis

Statistical Package for Social Sciences, version 20.0 Windows, (SPSS, and Chicago, IL) was used to perform data analysis. Data were cleaned and summarized. Statistical analyses included descriptive methods, including percentages of detection, serogroup distribution, and susceptibility to tetracycline, erythromycin, ciprofloxacin and azithromycin.

## Results

**Fig 1:**
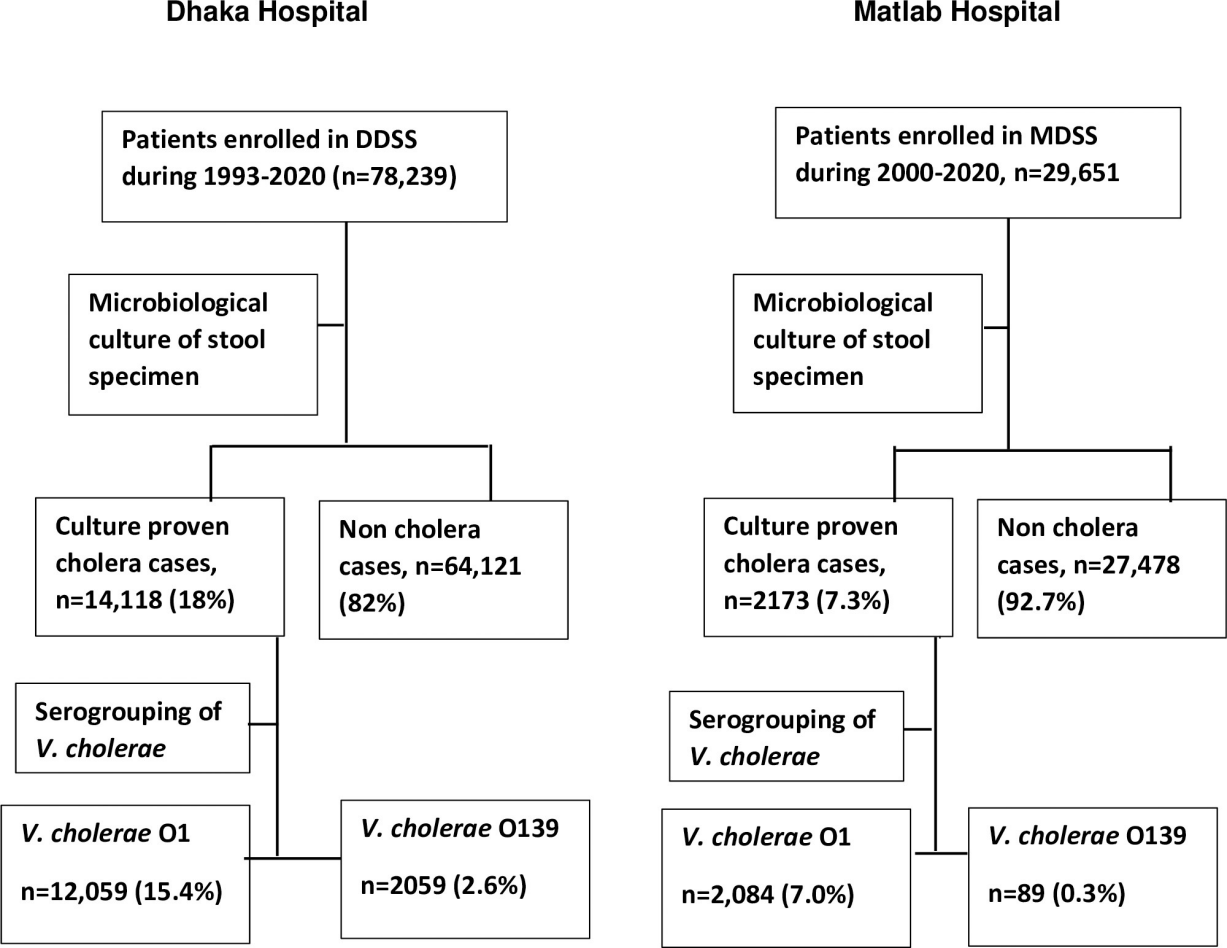
Study flowchart summarizing the selection processes of the study.

In the Dhaka Hospital, between 1^st^ January 1993 and 31^st^ December 2020, 78,239 diarrhoeal patients (irrespective of age and sex) were enrolled in the DDSS. Among them, 14,118 (18%) had microbiologically proven cholera. Two thousand and fifty-nine (2.6%) patients had cholera due to serogroup O139 among all the diarrhoeal patients (Fig 1). Based on the sample from surveillance system, the estimated total numbers of diarrhoea and cholera patients were 3,544,625 and 627,025, respectively over the period of 1993–2020.

At the Matlab Hospital, during the years 2000 to 2005, there were 34, 31, 15, 8, 0 and 1 isolates respectively of *Vibrio cholerae* O139 detected. Serogroup O139 was not detected at the Matlab Hospital since 2006 (Table 1).

**Table 1:** Yearly total *Vibrio cholerae* O139 and *Vibrio cholerae* O1 among diarrhoeal patients of Matlab Health and Demographic Surveillance System (HDSS) who attended to the Matlab Hospital of icddr,b; Bangladesh (2000–2020).

Year	*Vibrio cholerae* O139	*Vibrio cholerae* O1	Total patients
**2000**	34	73	1405
**2001**	31	116	1539
**2002**	15	173	1620
**2003**	8	153	1488
**2004**	0	319	1531
**2005**	1	181	1342
**2006**	0	74	1323
**2007**	0	105	1595
**2008**	0	63	1996
**2009**	0	114	1532
**2010**	0	206	1547
**2011**	0	48	1324
**2012**	0	68	1374
**2013**	0	29	1249
**2014**	0	86	1245
**2015**	0	62	1216
**2016**	0	27	1179
**2017**	0	25	1301
**2018**	0	51	1391
**2019**	0	94	1586
**2020**	0	17	868

**Fig 2:**
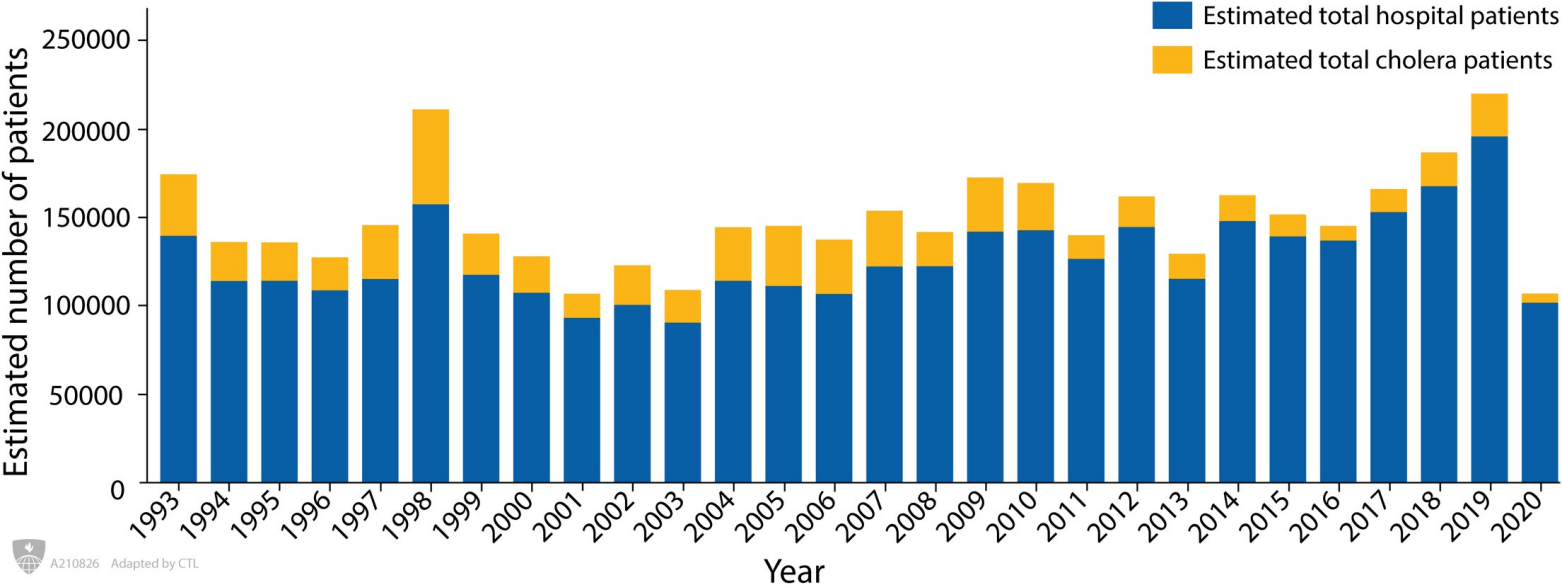
Yearly total number of estimated hospital patients and the total number of estimated cholera patients attended to the Dhaka Hospital of icddr,b; Bangladesh (1993–2020).

Fig 2 shows the yearly estimated number of hospital patients with diarrhoea and the estimated number of cholera cases between 1993 and 2020. During this period, the largest number of total cases occurred in 2019, whereas the largest number of cholera cases occurred in 1998; thereafter, the number of confirmed cholera cases declined. The lowest numbers of cholera cases were reported in 2020.

**Fig 3:**
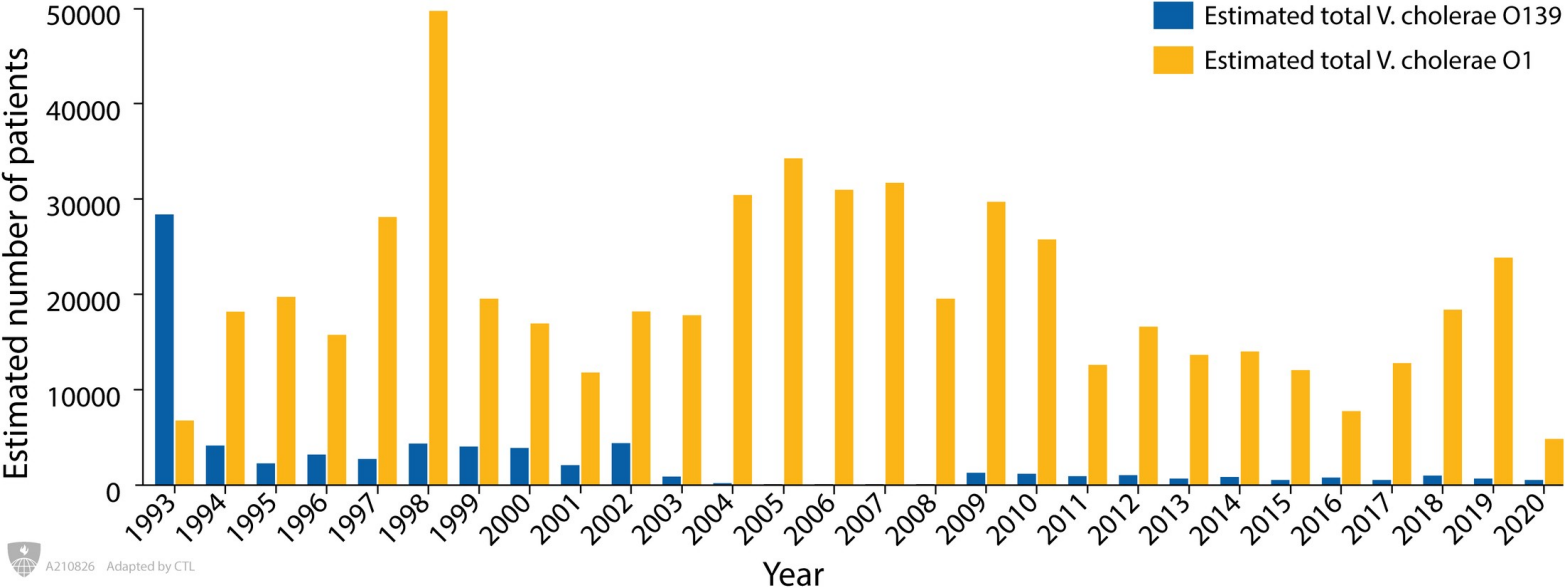
Yearly estimated total *Vibrio cholerae* O139 and *Vibrio cholerae* O1 among diarrhoeal patients attended to the Dhaka Hospital of icddr,b; Bangladesh (1993–2020).

Fig 3 shows the annual number of patients with cholera due to *V*. *cholerae* O139 and *V*. *cholerae* O1 during the study period. During 1993 *V*. *cholerae* O139 was the predominant serogroup of *V*. *cholerae*, but after this year, serogroup O1 predominated during rest of the study period. Between 2005 and 2008 there were no cases of *V*. *cholerae* O139 detected; but since 2009, there was a re-emergence of *V*. *cholerae* O139 and a limited number of cases were detected each year. During the last ten years (2011–2020), an average of thirteen *V*. *cholerae* O139 and an average of two hundred and seventy-one *V*. *cholerae* O1 were detected in the 2% surveillance sample.

Table 2 shows the baseline characteristics of the cholera patients by serogroup during a ten-year period between 2011 and 2020. A comparison of the two groups were generally similar except that, among cases with O139, a higher proportion of cases were in the youngest age group (p<0.05), maternal literacy was higher (p< 0.01) and drinking water was less frequently treated (p<0.05). For both groups, literacy rates were low and most used drinking water that was not treated.

**Table 2:** Baseline characteristics of the cholera patients by serogroup *Vibrio cholerae* O139 and *Vibrio cholerae* O1 attended to the Dhaka Hospital of icddr,b; Bangladesh (2011–2020).

Variables	*Vibrio cholerae* O139 (n = 134)	*Vibrio cholerae* O1 (n = 2711)	*p-value*
**Sex distribution by age**			
<5 years	49 (36.6%)	397 (14.6%)	**<0.05**
Female	18 (36.7%)	157(39.5%)	0.76
Male	31 (63.3%)	240(60.5%)	
5–14 years	09 (6.7%)	294 (10.8%)	0.17
Female	03 (33.3%)	101 (34.4%)	1.00
Male	06 (66.7%)	193 (65.6%)	
15+ years	76 (56.7%)	2020 (74.6%)	**<0.05**
Female	22 (28.9%)	827 (40.9%)	**0.04**
Male	54 (71.1%)	1193 (59.1%)	
**Area of residence**			
Other area	121 (90.3%)	2472 (91.2%)	0.64
Slum	13 (9.7%)	238 (8.8%)	
**Mother’s education**			
Literate	58 (43.3%)	887 (32.7%)	**0.01**
Illiterate	76 (56.7%)	1824 (67.3%)	
**Father’s education**			
Literate	59 (44.0%)	1005 (37.1%)	0.12
Illiterate	75 (56.0%)	1706 (62.9%)	
**Source of drinking water**			
Tap	97 (72.4%)	1908 (70.4%)	0.69
Non tap water	37 (27.6%)	803 (29.6%)	
**Type of drinking water**			
Boiled	30 (22.4%)	943 (34.8%)	**<0.05**
Not treated	104 (77.6%)	1768 (65.2%)	
**Type of toilet used**			
Sanitary/ semi sanitary	120 (89.6%)	2441 (90.0%)	0.88
Non sanitary	14 (10.4%)	270 (10.0%)	
**Wealth quintile**			
Rich	46 (34.6%)	994 (36.8%)	0.65
Upper middle	41 (30.8%)	805 (29.8%)	0.90
Middle	32 (24.1%)	574 (21.3%)	0.52
Lower middle	09 (6.8%)	252 (9.3%)	0.39
Poor	05 (3.8%)	74 (2.7%)	0.67

Table 3 shows the presenting features of cholera cases associated with *V*. *cholerae* O139 and *V*. *cholerae* O1 for patients treated between 2011 and 2020 Among both the groups, most cases presented within 24 hours of onset of watery diarrhoeal episode with some degree of dehydration. They reported using antibiotics commonly before coming to the hospital. Around two-thirds of the patients left the hospital within 24 hours of hospitalization. These presentations are typical of cholera as seen in Dhaka. Fewer patients infected with serogroup O139 had moderate or severe dehydration, vomiting, and required intravenous hydration. Nevertheless, most of these O139 patients were dehydrated and did require intravenous hydration. There were eight deaths among cholera patients between 1993 and 2020 including two between 2011 and 2020.

**Table 3:** Clinical characteristics of patients from whom *Vibrio cholerae* O139 and *Vibrio cholerae* O1 strains were isolated among those who attended to the Dhaka Hospital of icddr,b; Bangladesh (2011–2020).

Variables		*Vibrio cholera*e O139 (n = 134)	*Vibrio cholerae* O1 (n = 2711)	*p-value*
**Duration of diarrhoea before arrival**	<1 day	91 (67.9%)	1882 (69.4%)	0.79
	≥1 day	43 (32.1%)	829 (30.6%)	
**Character of stool**	Watery	130 (97.0%)	2680 (98.9%)	0.14
	Non-watery	04 (3.0%)	31 (1.1%)	
**Stool content**	No mucus or blood	122 (91.0%)	2539 (93.7%)	0.42
	Mucus	11 (8.2%)	167 (6.2%)	
	Blood	01 (0.7%)	01 (0.0%)	
**Number of stools in last 24 hours**	1–5	10 (7.5%)	122 (4.5%)	0.16
	6–10	124 (92.5%)	2589 (95.5%)	
**Vomiting in last 24 hours** *	No vomit	27 (20.1%)	296 (10.9%)	<0.05
	Vomit	107 (79.9%)	2415 (89.1%)	
**Abdominal pain**	No	60 (44.8%)	1105 (40.8%)	0.41
	Yes	74 (55.2%)	1606 (59.2%)	
**Temperature**	No fever	126 (94.0%)	2636 (97.2%)	0.06
	Had fever	08 (6.0%)	75 (2.8%)	
**Assessment of dehydration**	No dehydration	34 (25.4%)	149 (5.5%)	<0.05
	Some /Severe dehydration	100 (74.6%)	2562 (94.5%)	
**Use of replacement fluid before arrival**	None	11 (8.2%)	162 (06.0%)	0.38
	ORS at home	123 (91.8%)	2549 (94.0%)	
**Antibiotics before hospitalization** *	Used	67 (50.0%)	1712 (63.2%)	0.03
	Not used	67 (50.0%)	999 (36.8%)	
**Rehydration method used after hospitalization** *	ORS	72 (53.7%)	494 (18.2%)	<0.05
	Intravenous	62 (46.3%)	2217 (81.8%)	
**Duration of hospital stay**	<24 hours	48 (36.1%)	869 (32.1%)	0.39
	≥ 24 hours	85 (63.9%)	1834 (67.9%)	
**Outcome**	Cured/discharged	134 (100.0%)	2696 (88.9%)	NS
	Death	00 (0.0%)	02 (0.1%)	

* Significant at p<0.05

Because cholera is sometimes associated with concurrent infection with ETEC, we compared the proportion of the cases co-infected with ETEC. As shown in Table 4, ETEC were isolated from 8.1% to 13.8% of patients with serogroup O139 and 6.9% to 13.8% of patient with serogroup O1.

**Table 4:** Co-infections of *Vibrio cholerae* 0139 and enterotoxigenic E coli among diarrhoeal patients attending the Dhaka Hospital of icddr,b, Bangladesh (1996–2020).

Years	*V*. *cholerae* O139	*V*. *cholerae* O139 and ETEC	Rate of co-infection, O139 and ETEC	*V*. *cholerae* O1	*V*. *cholerae* O1 and ETEC	Rate of co-infection, O1 and ETEC
1996–2001	395	32	8.1%	2825	195	6.9%
2002–2008	ETEC were not tested 2002–2006 and *V*. *cholerae* O139 not detected 2005–2008					
2009–2014	109	15	13.8%	2234	197	8.8%
2015–2020	64	8	12.5%	1582	218	13.8%

The isolates of *V*. *cholerae* O139 were examined for susceptibility to tetracycline, erythromycin, ciprofloxacin, and azithromycin. Nearly all isolates were susceptible to tetracycline during 1990s and early 2000s; thereafter, some isolates were found to be resistant (Table 5). Most isolates were susceptible to erythromycin between 1993 and 2004, but when O139 re-emerged in 2009, the isolates that appeared were resistant to erythromycin during the following years. The susceptibility of *V*. *cholerae* O139 to ciprofloxacin and azithromycin was high, with some exceptions as shown on Table 5.

**Table 5:** Percentage of *V*. *cholerae* O139 susceptible to tetracycline, erythromycin, ciprofloxacin and azithromycin during the period, 1993 to 2020.

Year	Number of isolates tested	Tetracycline	Erythromycin	Ciprofloxacin	Azithromycin
1993–1998	1580	93.7%	94.3%		
1999–2004	299	98.7%	99.0%		
2005–2008	*V*. *cholerae* O139 was not isolated during this period				
2009–2014*	109*	78.0%	2.8%	93.6%	85.9%
2015–2020	71	64.8%	1.4%	90.1%	78.9%

*Azithromycin was not tested in 2009; n = 85 for azithromycin during 2010–2014

## Discussion

We report here the isolation of *V*. *cholerae* serogroup O139 in Dhaka, Bangladesh over a period of 28 years between January 1993 and December 2020. After the cholera epidemic of 1992–93, *V*. *cholerae* O139 when this serogroup predominated, it was then isolated much less frequently. Except for a period during 2005 to 2008, it has continued to cause cholera in urban Dhaka, but cases have not been detected in rural Matlab since 2006. The clinical disease associated with serogroup O139 is similar to cholera caused by serogroup O1; however, fewer of the O139 cases had severe dehydration. Nevertheless, most cases of cholera with O139 did have moderate or severe dehydration and required intravenous rehydration.

Treatment of cholera patients includes both immediate hydration with intravenous and/or oral rehydration solution as appropriate for the degree of dehydration, and the use of appropriate antibiotics. Antibiotics are needed to shorten the illness, reduce time in hospital, reduce the stool volume, and limit the fecal excretion of *V*. *cholerae* to prevent onward transmission of the infection [6, 15]. To treat effectively, the selection of antibiotic should match the susceptibility of *V*. *cholerae*. Though there had been strains resistant to tetracycline, most remained susceptible to tetracycline, ciprofloxacin, and azithromycin. Interestingly, strains of O139 were sensitive to erythromycin during the early years of the study period, but the strains that emerged after a four-year gap were resistant.

*V*. *cholerae* O139 is rarely isolated now in other parts of the Indian subcontinent. In Kolkata, very few isolates of O139 at the National Institute of Cholera and Enteric Diseases were detected since 2008; the last one in 2018 (personal communication Dr. Asish Mukhopadhyay). O139 has also been seen in Odisha during the last decade. A study conducted in Odisha, India between 2004–2013, found that *V*. *cholerae* O139 was not detectable between 2004 and 2005, but then it briefly re-emerged in 2006 [16]. Also in Odisha, after an interval of 10 years, *V*. *cholerae* O139 re-emerged during April and May 2017 following a major flood event [8]. The intermittent resurgence is remarkable and suggests that this strain may reappear in a region following a long period when it had apparently disappeared.

As reported earlier, children have higher rates of O1 cholera compared to adults but O139 cholera was reported to be more evenly distributed across the age spectrum [5, 17]. The lower rates of O1 cholera among older ages was thought to be due to acquired immunity in endemic areas, but since O139 is not endemic, there is less likelihood of developing immunity with age. Thus, it was somewhat surprising to observe higher numbers of O139 cholera in this study in which 36.6% of the cases occurred in children <5 years. This is considerably higher than expected since children <5 make up only 13.5% of the population. This study also showed a trend for patients infected with serogroup O139 coming from families with lower literacy rates.

Our results showed that cholera remains persistently in Dhaka causing large numbers of persons to become severely ill, requiring hospitalization. There were eight cholera deaths recorded during this study period including two occurring during the last ten years; however, other deaths may have occurred among patients who did not reach the hospital in time and were not counted. While cholera deaths are extremely rare among patients who reach the icddr,b hospital, it can be significantly higher in remote areas and among patients who are delayed in obtaining care or do not have access to medical care [18–20].

This study had some limitations. This hospital-based surveillance was conducted at two hospitals in Bangladesh and may not represent the situation in other areas of Bangladesh. Secondly, we did not carry out genomic studies to show that the isolates were toxigenic, nor did we measure antibody responses that are expected to occur in patients with cholera. Of interest, more of the patients with *V*. *cholerae* O139 were not dehydrated suggesting that these patients might have ingested a low inoculum or that the recovered isolates may not have been pathogenic. We did not rule out the possibility that some of these illnesses were caused by other pathogens and that the *V*. *cholerae* O139 represented an environmental organism passing through the gut. The most likely “other pathogen” that might cause this illness is ETEC, and we found that a small proportion of these cholera patients were co-infected with ETEC, but these co-infections occurred in patients with both serogroups. Though some did not show signs of moderate or severe dehydration, it should be noted that most patients with O139 cholera did have moderate or severe dehydration and a majority required intravenous rehydration. Also, these cases are consistent with the World Health Organization definition of a cholera case as a patient with acute watery diarrheal disease from whom *V*. *cholerae* O1 or O139 is detected. Additional genomic studies will be helpful in documenting their enterotoxicity and to compare other attributes of recent strains with earlier strains, especially those isolated before the gap years when erythromycin resistance appeared [21].

Rapid diagnostic tests (dipsticks) for cholera are being used increasingly to detect cholera. Some of these dipsticks have a line for O1 and another for O139, but other tests only have a line only for O1. In many areas, O139 has not been found and the rapid test which detects only O1 is felt to be preferable [22]; however, this will need to be reviewed in the future to determine if the test needs to detect both serogroups, especially if O139 is found to be spreading outside of Asia.

Future studies are needed to understand risk factors for O139 and the possibility that these cases are occurring in clusters or from specific locations in the Dhaka area where these infections are being transmitted. Surveillance needs to continue to identify changes in antimicrobial susceptibility patterns to inform treatment decisions. The observation that O139 strains seem to disappear for many years and then reappear suggests that strains may be emerging from environmental reservoir; thus, future studies are needed to determine if such an environmental niche exists. Finally, current WHO prequalified oral cholera vaccines are bivalent for both serogroups; however, protection against 0139 cholera has not been determined because cholera due to O139 was not reported in the field areas when the vaccine was evaluated. Since this vaccine has been administered to many people in Dhaka [23], it may be possible to determine if the vaccine protects against O139 disease by conducting case control studies.

In conclusion, cholera due to *V*. *cholerae* serogroup O139 continues to cause severe diarrhoea in urban Dhaka and the consistency of these ongoing illnesses seems to be unique among areas with cholera.
